# Evolutionary Dynamics of Microsatellite Distribution in Plants: Insight from the Comparison of Sequenced *Brassica*, *Arabidopsis* and Other Angiosperm Species

**DOI:** 10.1371/journal.pone.0059988

**Published:** 2013-03-28

**Authors:** Jiaqin Shi, Shunmou Huang, Donghui Fu, Jinyin Yu, Xinfa Wang, Wei Hua, Shengyi Liu, Guihua Liu, Hanzhong Wang

**Affiliations:** 1 Key Laboratory of Oil Crop Biology of the Ministry of Agriculture, Oil Crops Research Institute of the Chinese Academy of Agricultural Sciences, Wuhan, China; 2 Key Laboratory of Crop Physiology, Ecology and Genetic Breeding, Ministry of Education, Agronomy College, Jiangxi Agricultural University, Nanchang, China; Virginia Tech, United States of America

## Abstract

Despite their ubiquity and functional importance, microsatellites have been largely ignored in comparative genomics, mostly due to the lack of genomic information. In the current study, microsatellite distribution was characterized and compared in the whole genomes and both the coding and non-coding DNA sequences of the sequenced *Brassica*, *Arabidopsis* and other angiosperm species to investigate their evolutionary dynamics in plants. The variation in the microsatellite frequencies of these angiosperm species was much smaller than those for their microsatellite numbers and genome sizes, suggesting that microsatellite frequency may be relatively stable in plants. The microsatellite frequencies of these angiosperm species were significantly negatively correlated with both their genome sizes and transposable elements contents. The pattern of microsatellite distribution may differ according to the different genomic regions (such as coding and non-coding sequences). The observed differences in many important microsatellite characteristics (especially the distribution with respect to motif length, type and repeat number) of these angiosperm species were generally accordant with their phylogenetic distance, which suggested that the evolutionary dynamics of microsatellite distribution may be generally consistent with plant divergence/evolution. Importantly, by comparing these microsatellite characteristics (especially the distribution with respect to motif type) the angiosperm species (aside from a few species) all clustered into two obviously different groups that were largely represented by monocots and dicots, suggesting a complex and generally dichotomous evolutionary pattern of microsatellite distribution in angiosperms. Polyploidy may lead to a slight increase in microsatellite frequency in the coding sequences and a significant decrease in microsatellite frequency in the whole genome/non-coding sequences, but have little effect on the microsatellite distribution with respect to motif length, type and repeat number. Interestingly, several microsatellite characteristics seemed to be constant in plant evolution, which can be well explained by the general biological rules.

## Introduction

Microsatellites, which are also known as simple sequence repeats (SSRs), variable numbers of tandem repeats (VNTRs) and short tandem repeats (STRs, often defined as 1–6 bp), have been found in virtually all genomic regions (genic and non-genic regions) of all examined organisms [1], [2], [3]. Microsatellites are unstable genomic elements that have historically been designated as nonfunctional “junk DNA” and are mainly used as “neutral” genetic markers [3]. Recently, a large number of studies have shown that microsatellites can play many important biological functions (e.g., regulation of chromatin organization, DNA metabolic processes, gene activity and RNA structure) that are determined by their locations, and mutations in microsatellites may lead to functional variability [1], [4], [5] and ultimately phenotypic flexibility/plasticity for adaptation and evolution [2], [6], [7]. Therefore, microsatellites have emerged as the third major class of genetic variations [2], alongside single nucleotide polymorphisms (SNPs) and copy number variations (CNVs).

Despite their ubiquity and functional importance, microsatellites have largely been ignored in comparative genomics [2], and their evolutionary dynamics are poorly understood. Although several microsatellite distribution characteristics have been investigated in several sequenced plant species [8], [9], [10], [11], no definitive conclusions have been made. First, the software, algorithms and search parameters [12] used for the identification of microsatellites have differed across reports [13], which has made it difficult to compare and integrate these results. In addition, the physical positions of microsatellites were not analyzed in these previous studies, and the genomic distributions of microsatellites have been poorly characterized. More importantly, due to the lack of genomic information, a small number of plant species were analyzed in each of these previous reports [14], [15], and the evolutionary dynamics of microsatellite distribution in plants have therefore not yet been investigated.

Owing to the rapid development of high-throughput sequencing technology, the genomes of *Brassica rapa* [16], *Brassica oleracea* (data submitted) and *Brassica napus* (http://oilcrops.info:8080/; our unpublished data) have recently been sequenced by our own and several other institutes. Up to the present, the genome sequences of approximately 40 plant species (mainly angiosperms) are available in public databases (http://www.phytozome.net; http://genomevolution.org/wiki/index.php/Sequenced_plant_genomes). Polyploidy has played a major role in the evolution of many eukaryotes [17] and approximately 70% of all angiosperms have experienced one or more episodes of polyploidy [18]. Of these sequenced plant species, the five *Brassicaceae* family species represent classical examples of polyploidy: the allotetraploid species *B. napus* (AACC, 2n = 38) originated from a chromosome doubling event after the recent (∼ 0.01 MYA) natural hybridization between two diploid species *B. rapa* (AA, 2n = 20) and *B. oleracea* (CC, 2n = 18) [19], which both originated after a whole-genome triplication event from a common ancestor with a basic genome similar to that of *Arabidopsis thaliana* and *Arabidopsis lyrata* [20], [21]. Specifically, *B. rapa* and *B. oleracea* diverged approximately 5 MYA, *A. thaliana* and *A. lyrata* diverged approximately 10 MYA, and the *Arabidopsis* and *Brassica* genera diverged approximately 20 MYA [20], [21]. Dicots diverged from a common ancestor with monocots approximately 200 MYA [22]. Therefore, genomic changes associated with polyploidy and evolution can be investigated using comparative genomics between *B. napus*, *B. rapa*/*B. oleracea*, *A. thaliana*/*A. lyrata* and other sequenced angiosperm species [23], [24].

In the current study, microsatellite distribution was characterized in the whole genomes and both the coding and non-coding DNA sequences of recently sequenced *Brassica* species and compared to the closely related *Arabidopsis* and other sequenced angiosperm species to study their evolutionary dynamics in plants.

## Results

### Frequency of Microsatellites in Sequenced *Brassica* and Other Angiosperm Species

**Figure 1:**
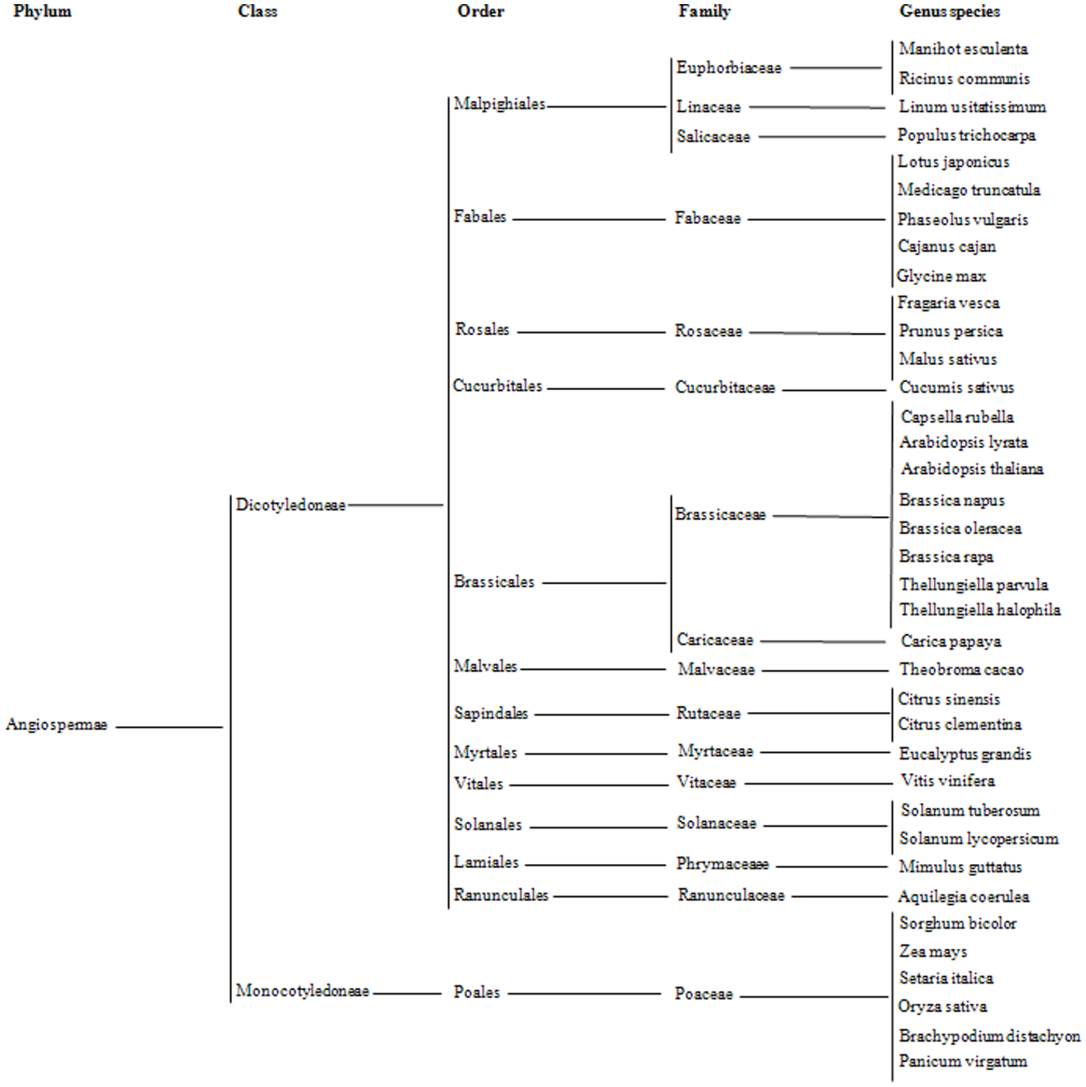
Taxonomic classification of the sequenced angiosperm species.

A total of 7, 974,520 microsatellites were identified in 18,503.1 Mb of assembled genomic sequences (CDSs+non-CDSs) from the sequenced *Brassica* and other angiosperm species (Table 1), which belong to two classes, thirteen orders, sixteen families and thirty-one genera (Figure 1). The variation in the microsatellite frequencies (3.7-fold) of these angiosperm species was much smaller than those for their microsatellite numbers (9.5-fold) and genome sizes (17.3-fold). Interestingly, the angiosperm species with large genome sizes (such as *Zea mays* and *Panicum virgatum*) and/or high transposable elements (TEs) contents (such as *Zea mays* and *Sorghum bicolor*) generally have a low or moderate microsatellite frequency, which was consistent with the significantly negative correlations between microsatellite frequencies and both genome sizes and TEs contents of these angiosperm species (r = −0.47 and −0.64, respectively).

**Table 1:** Number and frequency of microsatellites in the whole genomes and both the coding and non-coding DNA sequences of the sequenced *Brassica* and other angiosperm species.

Species	Whole genome					Non-coding DNA sequences				Coding DNA sequences			
	TEs	C/G	Sequence	SSRs	SSRs	C/G	Sequence	SSRs	SSRs	C/G	Sequence	SSRs	SSRs
	(%)	(%)	size (Mb)	number	frequency	(%)	size (Mb)	number	frequency	(%)	size (Mb)	number	frequency
*P. virgatum*	/	46.5%	1,358.1	454,339	334.5	46.1%	1,286.3	427,676	332.5	54.1%	71.8	26,663	371.6
*B. distachyon*	28.1%	46.4%	271.9	98,242	361.3	45.1%	230.5	82,623	358.4	53.4%	41.4	15,619	377.3
*O. sativa*	25.0%	43.6%	373.7	213,110	570.3	41.7%	318.4	173,908	546.1	54.2%	55.3	39,202	709.2
*S. italica*	46.3%	46.1%	405.7	120,895	298.0	45.1%	359.7	103,235	287.0	54.3%	46.1	17,660	383.4
*Z. mays*	85.0%	46.9%	2,065.7	464,899	225.1	46.6%	2,002.3	437,705	218.6	55.0%	63.4	27,194	428.9
*S. bicolor*	62.5%	45.3%	738.5	247,315	334.9	44.8%	701.5	228,265	325.4	54.8%	37.1	19,050	514.0
*A. coerulea*	/	36.9%	302.0	167,897	556.0	36.4%	268.8	159,082	591.8	41.3%	33.2	8,815	265.9
*M. guttatus*	/	35.5%	321.7	269,256	836.9	34.3%	288.5	258,009	894.3	46.2%	33.2	11,247	338.6
*S. lycopersicum*	/	35.5%	781.3	268,134	343.2	35.2%	744.3	260,211	349.6	41.7%	37.0	7,923	213.9
*S. tuberosum*	62.0%	34.8%	727.2	256,664	352.9	34.2%	676.3	247,282	365.6	42.5%	50.9	9,382	184.3
*V. vinifera*	21.5%	34.5%	486.2	325,204	668.9	33.9%	456.2	321,064	703.7	44.6%	30.0	4,140	138.2
*E. grandis*	/	39.3%	691.3	370,797	536.4	38.4%	630.5	356,973	566.2	48.1%	60.8	13,824	227.2
*C. clementina*	/	34.5%	295.6	190,487	644.5	32.9%	250.5	182,484	728.4	43.5%	45.0	8,003	177.8
*C. sinensis*	/	34.8%	319.2	141,396	442.9	32.8%	261.8	131,650	502.9	43.5%	57.4	9,746	169.7
*T. cacao*	24.0%	34.2%	327.4	133,264	407.1	32.8%	274.0	125,959	459.7	41.4%	53.3	7,305	136.9
*C. papaya*	52.0%	34.9%	342.7	182,323	532.1	34.2%	317.9	177,078	557.0	44.4%	24.8	5,245	211.5
*T. halophila*	/	37.7%	243.1	100,714	414.3	36.4%	207.0	91,666	442.9	45.2%	36.1	9,048	250.4
*T. parvula*	7.5%	35.9%	123.6	49,357	399.3	32.5%	91.4	42,129	460.7	45.6%	32.2	7,228	224.8
*B. napus*	/	36.8%	1,202.3	464,682	386.5	35.9%	1,099.0	435,179	396.0	46.3%	103.4	29,503	285.4
*B. rapa*	39.5%	35.3%	283.8	140,993	496.7	33.0%	235.7	127,253	539.9	46.3%	48.1	13,740	285.5
*B. oleracea*	43.0%	36.6%	540.0	229,389	424.8	35.6%	492.5	216,483	439.5	46.3%	47.5	12,906	272.0
*A. thaliana*	23.7%	36.1%	119.7	57,148	477.6	31.4%	76.1	46,471	610.5	44.1%	43.5	10,677	245.2
*A. lyrata*	29.7%	36.1%	206.7	100,424	485.9	34.4%	171.3	91,873	536.5	44.3%	35.4	8,551	241.4
*C. rubella*	/	35.6%	134.8	84,277	625.0	32.5%	99.2	74,919	755.4	44.5%	35.7	9,358	262.4
*C. sativus*	14.8%	32.4%	203.1	129,317	636.8	29.7%	162.4	121,512	748.2	43.4%	40.7	7,805	192.0
*M. domestica*	42.4%	38.0%	881.3	395,416	448.7	37.3%	810.1	381,932	471.4	46.1%	71.1	13,484	189.6
*P. persica*	/	37.5%	227.3	142,413	626.7	36.2%	192.5	136,131	707.2	44.6%	34.8	6,282	180.8
*F. vesca*	22.0%	38.0%	220.2	106,475	483.5	36.2%	179.8	97,298	541.1	46.0%	40.4	9,177	227.2
*G. max*	59.0%	34.8%	973.3	461,964	474.6	34.0%	905.1	446,894	493.8	44.2%	68.3	15,070	220.7
*C. cajan*	51.7%	32.8%	605.8	359,582	593.6	31.1%	559.1	351,996	629.6	53.2%	46.7	7,586	162.4
*P. vulgaris*	52.0%	34.2%	486.9	189,396	389.0	33.3%	446.8	182,327	408.1	43.8%	40.1	7,069	176.5
*M. truncatula*	30.0%	33.2%	307.5	153,053	497.8	32.1%	268.4	144,576	538.7	40.9%	39.1	8,477	216.7
*L. japonicus*	22.5%	37.3%	316.9	121,931	384.8	36.7%	287.4	114,513	398.5	44.0%	29.5	7,418	251.5
*P. trichocarpa*	35.0%	33.6%	417.1	269,242	645.5	32.3%	365.3	259,515	710.4	43.4%	51.8	9,727	187.7
*L. usitatissimum*	/	39.6%	318.3	105,277	330.8	37.9%	266.1	89,559	336.6	48.0%	52.2	15,718	301.1
*R. communis*	50.3%	33.8%	350.6	175,525	500.6	32.8%	319.3	169,119	529.7	44.8%	31.3	6,406	204.4
*M. esculenta*	/	35.5%	532.5	233,723	438.9	34.9%	492.5	227,215	461.3	43.3%	40.0	6,508	162.7
Total/Mean	38.7%	37.3%	18,503.1	7,974,520	475.8	36.0%	16,794.5	7,521,764	512.0	46.3%	1708.5	452,756	259.2
Variations (fold)	11.3	1.4	17.3	9.4	3.7	1.6	26.3	10.6	4.1	1.3	4.2	9.5	5.2

**Figure 2:**
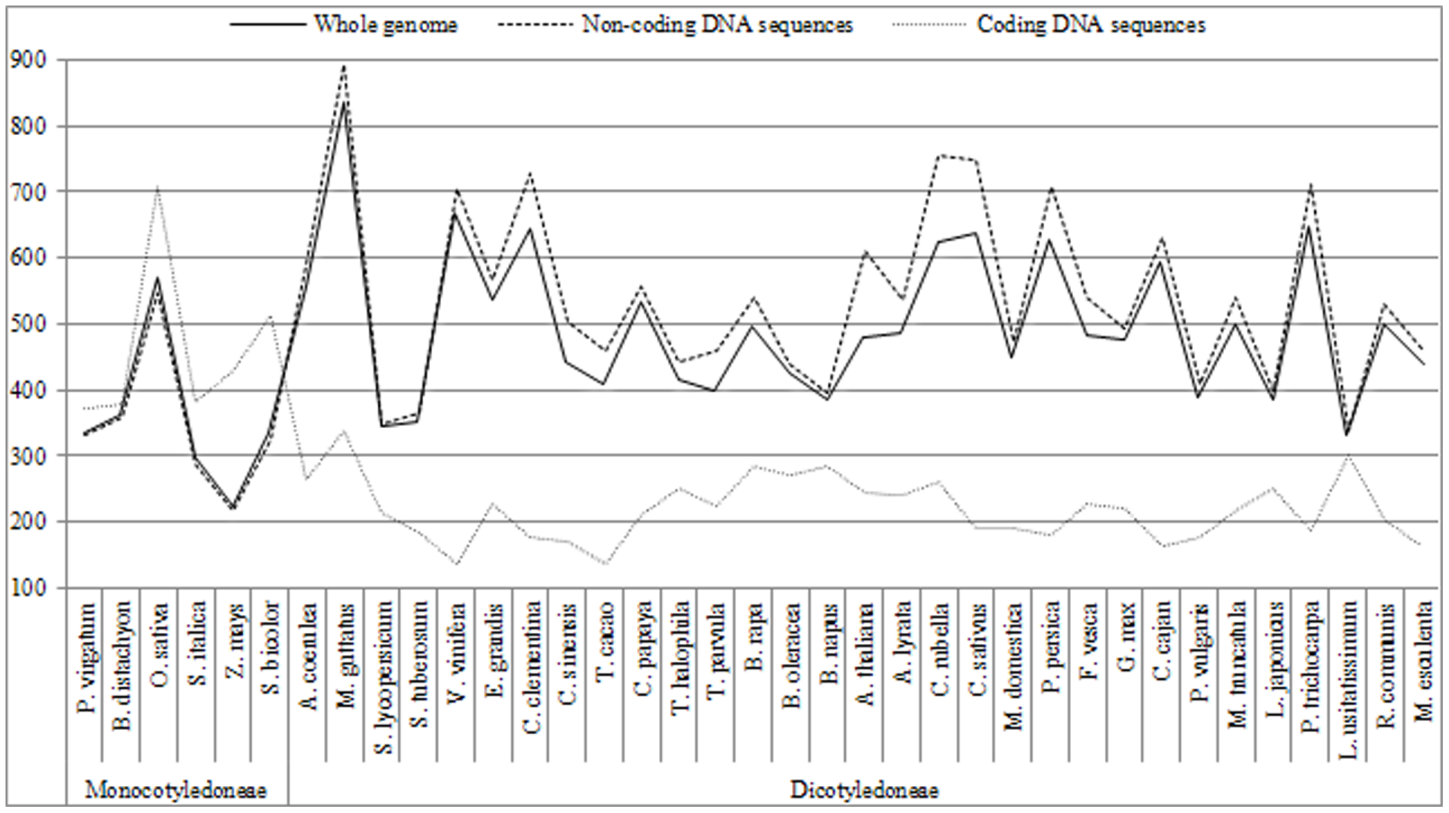
Microsatellite frequencies in the whole genome (solid line) and both the coding (dashed line of circular points) and non-coding (dashed line of square points) sequences of the sequenced *Brassica* and other angiosperm species. The horizontal axis displays the scientific names of these sequenced angiosperms in phylogenetic order. The vertical axis indicates the frequencies of microsatellites.

The microsatellite frequencies of the species within the same genus (such as *Brassica*) were generally comparable for the whole genome/non-coding sequences and similar for the coding sequences (Figure 2). However, when comparisons were conducted between species over a large phylogenetic distance, the differences in microsatellite frequencies usually became more pronounced for the whole genomes and both the coding and non-coding sequences. For example, the difference between the average microsatellite frequencies of the species of the *Monocotyledoneae* and *Dicotyledoneae* classes was significant for the whole genome and both the coding and non-coding sequences, whereas those differences between the *Brassica* and *Arabidopsis* genera and between the *Brassicales* and *Fabales* orders were significant only for the coding sequences (Table 2). In addition, the differences between the average microsatellite frequencies in the whole genomes and both the coding and non-coding sequences of the species of the *Monocotyledoneae* and *Dicotyledoneae* classes were all greater than those between the *Brassicales* and *Fabales* orders and were also greater than those between the *Brassica* and *Arabidopsis* genera.

**Table 2:** Comparison of the frequencies of microsatellites in the whole genomes and the coding and non-coding DNA sequences of the sequenced *Brassica* and other angiosperm species.

	*Brassicaceae*				*Brassicales*			*Dicotyledoneae*				*Angiospermae*			
	*Brassica*	*Arabidopsis*	Difference	Pt-test	*Brassicaceae*	*Caricaceae*	Difference	*Brassicales*	*Fabales*	Difference	Pt-test	*Dicotyledoneae*	*Monocotyledoneae*	Difference	Pt-test
**Whole genome**	436.0	481.7	−45.7	6.2E−01	463.8	532.1	−68.3	471.4	467.9	3.4	9.4E−01	499.4	354.0	145.4	**8.1E**−**03**
**Non-coding DNA sequences**	458.5	573.5	−115.0	1.6E−01	522.7	557.0	−34.4	526.5	493.7	32.8	5.8E−01	544.4	344.7	199.7	**1.9E**−**03**
**Coding DNA sequences**	281.0	243.3	37.6	**3.7E**−**02**	258.4	211.5	46.8	253.2	205.6	47.6	**3.8E**−**02**	219.5	464.1	−244.6	**1.3E**−**09**

Typically, compared to the *Monocotyledoneae* species, the average microsatellite frequency of the *Dicotyledoneae* species was significantly higher for the whole genome and the non-coding sequences (ratio = 1.41 and 1.58) but much lower for the coding sequences (ratio = 0.47).

Significant difference was also observed between the frequencies of microsatellites in the coding and non-coding sequences of the angiosperm species (P_t-test_ = 1.4E^−11^). Compared to the non-coding sequences, microsatellite frequency in the coding sequences was not significantly higher for all the *Monocotyledoneae* species (mean ratio = 1.35; P_t-test_ = 0.12) but significantly lower for all the *Dicotyledoneae* species (mean ratio = 0.40; P_t-test_ = 7.2E^−15^). In addition, the microsatellite frequencies in the coding and non-coding sequences were highly positively correlated for the *Monocotyledoneae* species (r = 0.80) but not significantly correlated for the *Dicotyledoneae* species (r = 0.00).

### Distribution of Microsatellites with Respect to Motif Length in Sequenced *Brassica* and Other Angiosperm Species

**Figure 3:**
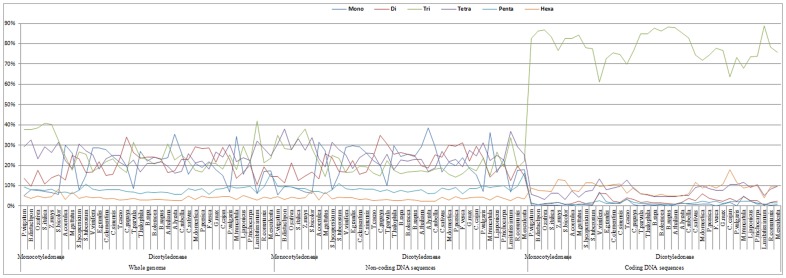
Microsatellites distribution with respect to motif length in the whole genomes and both the coding and non-coding regions of the sequenced *Brassica* and other angiosperm species. The horizontal axis displays the scientific names of these sequenced angiosperm species in phylogenetic order. The vertical axis indicates the relative abundances of the mono- to hexanucleotide repeats microsatellites. The colors of the legends indicate the length of the motifs from mono- to hexanucleotide.

The distributions of microsatellites with respect to motif length, i.e., the relative abundances of mono- to hexanucleotide repeat microsatellites, of the species within the same genus (such as *Brassica*) were generally very similar for the whole genome and non-coding sequences and almost identical for the coding sequences (Figure 3). However, in accordance with the general trend for the correlation of the abundance of the corresponding mono- to hexanucleotide repeats among these angiosperm species (i.e., the further the phylogenetic distance, the smaller the correlation coefficients; Table S1A-B), the differences in these variables generally became larger as the phylogenetic distance increased, for the coding sequences and especially the non-coding sequences and the whole genome (Table 3). For example, in the whole genome, the coding sequences and the non-coding sequences, the numbers (1, 0 and 2, respectively) of the types of microsatellite repeats (from mono- to hexanucleotide) that displayed significantly different abundances between the species of the *Brassica* and *Arabidopsis* genera were all less than those (3, 4 and 3, respectively) between the *Brassicales* and *Fabales* orders and were also less than those (5, 4 and 4, respectively) between the *Monocotyledoneae* and *Dicotyledoneae* classes. In addition, the differences between the average abundances of the individual mono- to hexanucleotide repeats in the whole genome, the coding and non-coding sequences of the species of the *Brassica* and *Arabidopsis* genera were usually smaller than those between the *Brassicales* and *Fabales* orders and were also smaller than those between the *Monocotyledoneae* and *Dicotyledoneae* classes.

**Table 3:** Comparison of the abundance of the individual mono- to hexanucleotide repeat microsatellites in the whole genomes and both the coding and non-coding DNA sequences of the sequenced *Brassica* and other angiosperm species.

	Motif length	*Brassicaceae*				*Brassicales*			*Dicotyledoneae*				*Angiospermae*			
		*Brassica*	*Arabidopsis*	Differncece	Pt-test	*Brassicaceae*	*Caricaceae*	Differncece	*Brassicales*	*Fabales*	Differncece	Pt-test	*Dicotyledoneae*	*Monocotyledoneae*	Differncece	Pt-test
**Whole genome**	**Mono**	23.1%	29.7%	−6.6%	4.5E−01	23.8%	19.5%	4.3%	23.3%	18.0%	5.3%	3.4E−01	21.0%	6.8%	14.2%	**5.2E−05**
	**Di**	23.8%	16.9%	7.0%	**1.3E**−**02**	22.3%	34.0%	−11.8%	23.6%	20.8%	2.8%	4.0E−01	21.3%	13.5%	7.8%	**2.2E**−**03**
	**Tri**	21.9%	26.7%	−4.8%	4.3E−01	24.9%	16.5%	8.4%	24.0%	22.4%	1.6%	5.7E−01	22.8%	37.9%	−15.0%	**9.1E**−**08**
	**Tetra**	21.3%	17.8%	3.4%	3.1E−01	19.5%	19.6%	−0.1%	19.5%	25.4%	−5.9%	**1.1E**−**02**	23.0%	28.7%	−5.7%	**3.1E**−**03**
	**Penta**	6.8%	6.2%	0.6%	4.4E−01	6.5%	7.1%	−0.7%	6.5%	8.9%	−2.4%	**5.0E**−**05**	8.0%	8.0%	0.0%	9.7E−01
	**Hexa**	3.1%	2.8%	0.4%	2.2E−01	3.0%	3.2%	−0.2%	3.1%	4.4%	−1.4%	**1.1E**−**02**	3.9%	5.1%	−1.3%	**1.0E**−**02**
**Non-coding DNA sequences**	**Mono**	24.9%	33.9%	−9.1%	3.0E−01	26.4%	20.1%	6.3%	25.7%	18.8%	7.0%	2.4E−01	22.3%	7.7%	14.6%	**8.9E**−**11**
	**Di**	25.6%	19.4%	6.3%	**1.2E**−**02**	24.6%	34.9%	−10.3%	25.8%	21.5%	4.3%	2.0E−01	22.5%	15.1%	7.4%	**1.6E**−**03**
	**Tri**	16.7%	17.2%	−0.5%	3.4E−01	17.8%	14.7%	3.1%	17.5%	20.3%	−2.8%	2.6E−01	19.0%	31.8%	−12.7%	**2.6E**−**04**
	**Tetra**	22.6%	20.1%	2.5%	5.5E−01	21.2%	19.9%	1.3%	21.1%	26.1%	−5.0%	**2.4E**−**02**	24.1%	31.8%	−7.8%	**3.1E**−**03**
	**Penta**	7.3%	7.1%	0.2%	8.4E−01	7.1%	7.3%	−0.2%	7.1%	9.2%	−2.1%	**2.2E**−**04**	8.4%	8.9%	−0.5%	3.4E−01
	**Hexa**	3.0%	2.4%	0.5%	**2.6E**−**02**	2.7%	3.0%	−0.3%	2.8%	4.1%	−1.4%	**1.1E**−**02**	3.6%	4.6%	−1.0%	2.0E−01
**Coding DNA sequences**	**Mono**	0.5%	1.0%	−0.6%	4.1E−01	0.6%	0.7%	0.0%	0.6%	2.0%	−1.4%	1.6E−01	1.3%	0.3%	1.0%	**5.6E**−**04**
	**Di**	1.4%	1.4%	0.1%	9.1E−01	1.8%	3.2%	−1.4%	2.0%	3.2%	−1.2%	5.2E−02	2.7%	1.1%	1.6%	**2.5E**−**05**
	**Tri**	87.3%	86.6%	0.7%	6.9E−01	86.0%	76.5%	9.4%	84.9%	71.0%	13.9%	**1.6E**−**03**	77.7%	83.0%	−5.2%	**1.9E**−**02**
	**Tetra**	4.6%	4.9%	−0.3%	5.8E−01	5.0%	8.6%	−3.5%	5.4%	9.9%	−4.5%	**5.7E**−**04**	8.0%	4.9%	3.1%	**1.1E**−**03**
	**Penta**	0.9%	1.1%	−0.2%	1.3E−01	1.1%	1.9%	−0.9%	1.2%	2.0%	−0.9%	**7.1E**−**03**	1.6%	1.3%	0.3%	2.7E−01
	**Hexa**	5.3%	5.0%	0.3%	3.6E−01	5.4%	9.1%	−3.6%	5.9%	11.9%	−6.0%	**1.7E**−**02**	8.7%	9.5%	−0.7%	5.6E−01

Typically, the distribution of microsatellites with respect to motif length of the *Monocotyledoneae* and *Dicotyledoneae* (except for *Linum usitatissimum*) species (Figure 3) was clearly different for the whole genome and the non-coding sequences but generally similar for the coding sequences (Table S1A-B). In the whole genome and non-coding sequences: for the *Monocotyledoneae* species, tri- or tetranucleotide repeats were the most abundant and were followed in abundance by dinucleotide repeats, whereas penta-, mono- and hexanucleotide repeats were relatively uncommon; whereas, for the *Dicotyledoneae* species (except for *Linum usitatissimum*), mono-, di-, tri- and tetranucleotide repeats displayed comparable and relatively high proportions, whereas penta- and hexanucleotide repeats showed relatively low proportions (Figure 3). In the coding sequences: for both the *Monocotyledoneae* and *Dicotyledoneae* species, trinucleotide repeats were dominant and were followed in abundance by the hexa- and tetranucleotide repeats, whereas the di-, penta- and mononucleotide repeats were not commonly identified (Figure 3). Compare to the *Monocotyledoneae* species, the average abundance of microsatellites in the *Dicotyledoneae* species was significantly lower for the tri-, tetra- and hexanucleotide repeats in the whole genome, for the tri- and tetranucleotide repeats in the non-coding sequences and for the trinucleotide repeats in the coding sequences, but was significantly higher for the mono- and dinucleotide repeats in the whole genome/non-coding sequences and for the mono-, di- and tetranucleotide repeats in the coding sequences (Table 3).

Great differences were found between the distributions of microsatellites with respect to motif length in the coding and non-coding sequences of all the angiosperm species (Figure 3). Compared to the non-coding sequences, the average abundance of microsatellites in the coding sequences of all the angiosperm species was significantly higher for the tri- and hexanucleotide repeats but significantly lower for the mono-, di-, tetra- and pentanucleotide repeats (Table S1C). In addition, the correlations between the abundance of the mono- to hexanucleotide repeats in the coding and non-coding sequences of the angiosperm species were all not significant (mean r = 0.58 and 0.10 for the *Monocotyledoneae* and *Dicotyledoneae* species, respectively; Table S1D).

### Distribution of Microsatellites with Respect to Motif Type in Sequenced *Brassica* and Other Angiosperm Species

**Figure 4:**
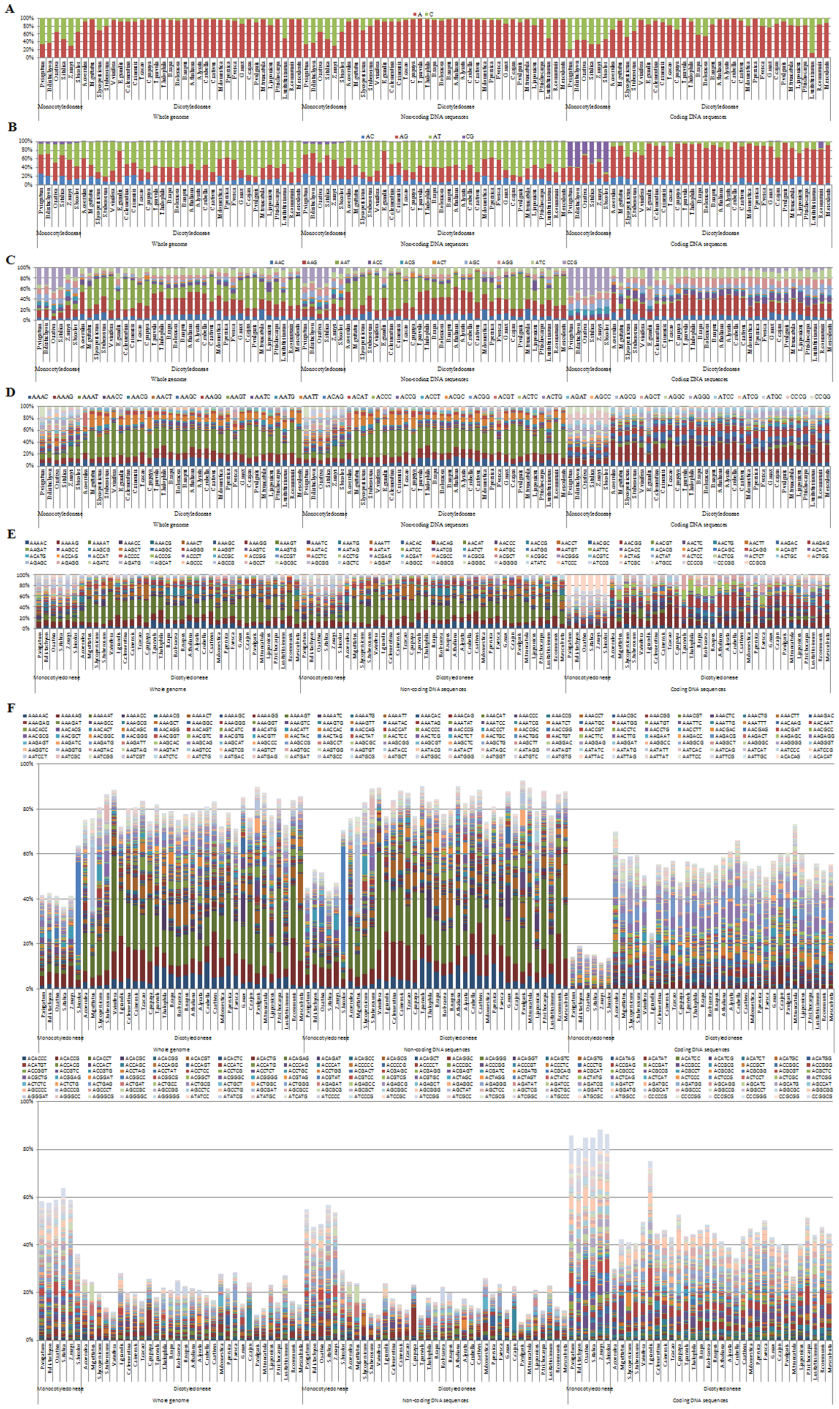
Distributions of microsatellites with respect to motif types in the whole genome and both the coding and non-coding sequences of the sequenced *Brassica* and other angiosperm species, for the individual mono- to hexanucleotide (A–F) repeats. The horizontal axis displays the scientific names of the analyzed plants with the sequenced genomes in phylogenetic order. The vertical axis indicates the relative proportions of the different motifs. The colors of legends indicate the type of the motifs.

The distributions of microsatellites with respect to motif type, i.e., the relative abundances of the mono- to hexanucleotide motifs, of the species within the same genus (such as *Brassica*) were highly similar for the whole genome and the non-coding sequences and nearly identical for the coding sequences (Figure 4A–F). However, in accordance with the general trend (i.e., the further the phylogenetic distance, the smaller the correlation coefficients) for the correlation of the corresponding abundance of all the mono- to hexanucleotide motifs among these angiosperm species (Table S2A–B), the differences in these variables generally increased as the phylogenetic distance increased, for the coding sequences and especially the non-coding sequences and the whole genome (Table S2C). For example, in the whole genomes, the coding sequences and the non-coding sequences, the numbers (62, 34 and 52, respectively) of the types of microsatellite motifs that displayed significantly different abundances between the species of the *Brassica* and *Arabidopsis* genera were all less than those (97, 51 and 71, respectively) between the *Brassicales* and *Fabales* orders and were also less than those (239, 282 and 239, respectively) between the *Monocotyledoneae* and *Dicotyledoneae* classes. In addition, the differences between the average abundances of the corresponding motifs in the whole genomes, the coding sequences, and the non-coding sequences of the species of the *Brassica* and *Arabidopsis* genera were usually smaller than those between the *Brassicales* and *Fabales* orders and were also smaller than those between the *Monocotyledoneae* and *Dicotyledoneae* classes.

Typically, in the whole genome and both the coding and non-coding sequences, the distribution of microsatellites with respect to motif type of the *Monocotyledoneae* and *Dicotyledoneae* species were clearly different (Figure 4). Compared to the *Monocotyledoneae* species, the average abundance of microsatellites in the whole genome/non-coding sequences and the coding sequences of the *Dicotyledoneae* species was significantly lower mostly and all for C/G-rich motifs but significantly higher mostly and more frequently for A/T-rich motifs, respectively (Table S2C), which corresponds the higher C/G contents in the sequenced *Monocotyledoneae* than *Dicotyledoneae* species (mean ratio = 1.28, 1.31 and 1.22 for whole genome, the non-coding sequences and the coding sequences, respectively; Table 1). Especially, both the dominant/major and absent/scarce motifs in the whole genome and both the coding and non-coding sequences of the *Monocotyledoneae* and *Dicotyledoneae* species were obviously different (Table 4). In the whole genome and non-coding sequences: for the *Monocotyledoneae* species, the dominant/major motifs were more frequently rich in A/T than C/G, and the absent/scarce motifs were basically equally rich in A/T and C/G (Table 4), which corresponds to their slightly higher A/T (mean = 54.2% and 55.1%) than C/G (mean = 45.8% and 44.9%,) content (Table 1); whereas, for the *Dicotyledoneae* species, the dominant/major motifs were all rich in A/T, and the absent/scarce motifs were all rich in C/G (Table 4), which corresponds to their much higher A/T (mean = 54.2% and 55.1%) than C/G (mean = 35.8% and 34.2%) content (Table 1). In the coding sequences: for the *Monocotyledoneae* species, the dominant/major motifs were all rich in C/G, and the absent/scarce motifs were all rich in A/T (Table 4), which corresponds to their slightly lower A/T (mean = 45.7%) than C/G (mean = 54.3%) content (Table 1); for the *Dicotyledoneae* species, the dominant/major motifs were mostly rich in A/T, and the absent/scarce motifs were more frequently rich in C/G than A/T (Table 4), which corresponds to their higher A/T (mean = 55.6%) than C/G (mean = 44.4%) content (Table 1).

**Table 4:** The dominant/major and absent/scarce motifs for the individual mono- to hexanucleotide repeats in the whole genomes and both the coding and non-coding DNA sequences of the sequenced *Monocotyledoneae* and *Dicotyledoneae* species.

	Class	Repeat type	Dominant/major motifs	Absent/scarce motifs
**Whole genome**	***Monocotyledoneae***	**Mono**	**/**	**/**
		Di	AG	CG
		Tri	CCG	ACT
		Tetra	AAAT	(A/T):(C/G) = 0.90∶1
		Penta	AAAAG, AAAAT	(A/T):(C/G) = 1.27∶1
		Hexa	AAAAAG, AACTAG	(A/T):(C/G) = 1.25∶1
	***Dicotyledoneae***	Mono	A	C
		Di	AT	CG
		Tri	AAT, AAG	ACG, CCG, ACT,AGC
		Tetra	AAAT, AAAG, AATT, AAAC	(A/T):(C/G) = 0.38∶1
		Penta	AAAAT, AAAAG, AAATT, AAAAC	(A/T):(C/G) = 0.47∶1
		Hexa	AAAAAT, AAAAAG, AAAAAC, AAAATT	(A/T):(C/G) = 0.71∶1
**Non-coding DNA sequences**	***Monocotyledoneae***	Mono	/	/
		Di	AG	CG
		Tri	CCG	ACT
		Tetra	AAAT	(A/T):(C/G) = 0.91∶1
		Penta	AAAAG, AAAAT	(A/T):(C/G) = 1.28∶1
		Hexa	AACTAG,AAAAAG	(A/T):(C/G) = 1.17∶1
	***Dicotyledoneae***	Mono	A	C
		Di	AT	CG,AC
		Tri	AAT, AAG	ACG,CCG,AGC,ACT
		Tetra	AAAT, AAAG, AATT,AAAC	(A/T):(C/G) = 0.61∶1
		Penta	AAAAT, AAAAG, AAATT, AAAAC	(A/T):(C/G) = 0.77∶1
		Hexa	AAAAAT, AAAAAG, AAAAAC, AAAATT	(A/T):(C/G) = 0.80∶1
**Coding DNA sequences**	***Monocotyledoneae***	Mono	/	/
		Di	CG	AT
		Tri	CCG	AAT, ACT, AAC, ATC
		Tetra	CCCG, CCGG, AGGG, AAAG	(A/T):(C/G) = 1.44∶1
		Penta	CCGCG	(A/T):(C/G) = 2.20∶1
		Hexa	CCGGCG, ACGGCG, AGGCGG	(A/T):(C/G) = 1.96∶1
	***Dicotyledoneae***	Mono	A	C
		Di	AG,	CG
		Tri	AAG	ACT, AAT, ACG,CCG
		Tetra	AAAG	(A/T):(C/G) = 1.00∶1
		Penta	AAAAG, AAGAG	(A/T):(C/G) = 1.14∶1
		Hexa	AAGAGG, AAGATG	(A/T):(C/G) = 1.32∶1

Obvious differences were found between the distribution of microsatellites with respect to motif type in the coding and non-coding sequences of all the angiosperm species (Figure 4). Compare to the non-coding sequences, the average abundance of microsatellites in the coding sequences of all the angiosperm species was significantly higher mostly for C/G-rich motifs but significantly lower mostly for A/T-rich motifs (Table S2D), which corresponds to their higher C/G content in the coding (46.3%) than non-coding (36.0%) sequences (Table 1). In addition, the correlations between the relative abundance of all the corresponding mono- to hexanucleotide motifs in the coding and non-coding sequences of the angiosperm species were generally moderate (mean r = 0.54 and 0.25 for the *Monocotyledoneae* and *Dicotyledoneae* species, respectively; Table S2E).

### Distribution of Microsatellites with Respect to Motif Repeat Number in Sequenced *Brassica* and Other Angiosperm Species

**Figure 5:**
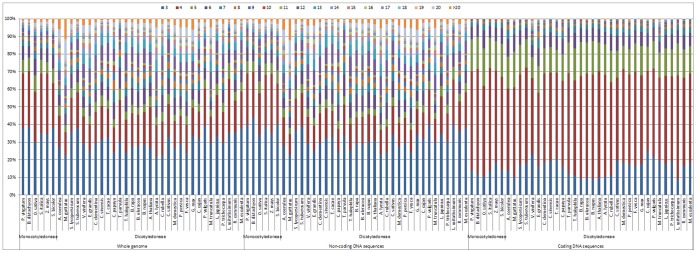
Microsatellites distribution with respect to motif repeat numbers (3 to 20, and >20) in the whole genome and both the coding and non-coding sequences of the sequenced *Brassica* and other angiosperm species. The horizontal axis displays the scientific names of the sequenced angiosperm species in phylogenetic order. The vertical axis indicates the relative abundances of the corresponding motif repeat numbers microsatellites. The colors of legends indicate the repeat number of motifs.

The distributions of microsatellites with respect to motif repeat number, i.e., the relative abundances of microsatellites of different motif repeat numbers, in the whole genomes, the non-coding sequences and especially in the coding sequences of the species of the same class (such as *Monocotyledoneae* or *Dicotyledoneae*) were almost identical (Figure 5). Whereas, in accordance with the relatively weak correlations between the abundance of microsatellites of the corresponding motif repeat numbers of the *Monocotyledoneae* and *Dicotyledoneae* species (Table S3A–B), the differences in these variables in the coding sequences and especially in whole genome and the non-coding sequences of the two classes were generally significant (Table 5; Table S3C). Compared to the *Monocotyledoneae* species, the average abundance of microsatellites of the *Dicotyledoneae* species was significantly lower for the 3–5 and 5 times of motif repeat but higher for >7 and >8 times of motif repeat, respectively, for the whole genome/non-coding sequences and the coding sequences.

**Table 5:** Comparison of the abundances of certain motif repeat number microsatellites in the whole genomes of the sequenced *Brassica* and other angiosperm species.

Motif repeat numbers	*Brassicaceae*				*Brassicales*			*Dicotyledoneae*				*Angiospermae*			
	*Brassica*	*Arabidopsis*	Difference	Pt-test	*Brassicaceae*	*Caricaceae*	Difference	*Brassicales*	*Fabales*	Difference	Pt-test	*Dicotyledoneae*	*Monocotyledoneae*	Difference	Pt-test
**3**	27.7%	24.2%	3.5%	3.6E−01	26.0%	23.9%	2.1%	25.7%	33.7%	−8.0%	**4.2E**−**03**	29.8%	36.0%	−6.3%	**3.3E**−**03**
**4**	18.2%	20.3%	−2.1%	5.7E−01	19.4%	13.9%	5.5%	18.8%	18.1%	0.7%	7.4E−01	18.2%	30.8%	−12.6%	**3.5E**−**05**
**5**	4.4%	5.2%	−0.8%	4.7E−01	5.1%	4.6%	0.4%	5.0%	5.1%	−0.1%	8.8E−01	5.2%	8.0%	−2.9%	**1.6E**−**04**
**6**	9.2%	7.2%	2.1%	1.7E−01	8.9%	9.4%	−0.5%	9.0%	6.4%	2.6%	**5.0E**−**03**	7.7%	7.6%	0.1%	9.2E−01
**7**	5.2%	3.6%	1.6%	**2.6E**−**02**	4.9%	6.7%	−1.8%	5.1%	3.7%	1.4%	**3.7E**−**02**	4.4%	3.6%	0.8%	**2.4E**−**02**
**8**	3.5%	2.4%	1.1%	**1.8E**−**02**	3.3%	5.8%	−2.5%	3.6%	2.7%	0.9%	1.3E−01	3.1%	2.0%	1.1%	**5.2E**−**05**
**9**	2.4%	1.7%	0.8%	**4.6E**−**03**	2.3%	4.8%	−2.5%	2.6%	2.0%	0.6%	2.4E−01	2.2%	1.2%	1.1%	**4.0E**−**07**
**10**	1.7%	1.2%	0.5%	**2.1E**−**02**	1.6%	3.4%	−1.9%	1.8%	1.5%	0.3%	4.9E−01	1.6%	0.7%	0.9%	**1.9E**−**08**
**11**	1.2%	0.8%	0.4%	**2.0E**−**02**	1.1%	2.3%	−1.3%	1.2%	1.1%	0.1%	8.0E−01	1.2%	0.4%	0.7%	**1.2E**−**09**
**12**	8.1%	8.6%	−0.4%	7.9E−01	8.3%	7.0%	1.2%	8.1%	6.8%	1.3%	2.2E−01	7.2%	2.6%	4.6%	**5.6E**−**15**
**13**	5.0%	5.6%	−0.6%	6.5E−01	5.0%	4.7%	0.3%	4.9%	4.3%	0.6%	3.9E−01	4.6%	1.6%	3.0%	**3.3E**−**14**
**14**	3.4%	4.1%	−0.8%	4.8E−01	3.5%	3.4%	0.1%	3.5%	3.0%	0.5%	4.2E−01	3.4%	1.0%	2.3%	**5.9E**−**14**
**15**	2.5%	3.3%	−0.8%	4.5E−01	2.7%	2.6%	0.0%	2.6%	2.4%	0.3%	5.7E−01	2.6%	0.8%	1.8%	**5.1E**−**13**
**16**	1.8%	2.6%	−0.8%	3.1E−01	2.0%	1.9%	0.0%	2.0%	1.9%	0.1%	8.2E−01	1.9%	0.6%	1.4%	**1.6E**−**11**
**17**	1.3%	2.0%	−0.7%	3.3E−01	1.4%	1.3%	0.1%	1.4%	1.4%	0.0%	9.5E−01	1.4%	0.4%	1.0%	**7.4E**−**10**
**18**	0.9%	1.5%	−0.6%	2.8E−01	1.0%	1.0%	0.1%	1.0%	1.1%	−0.1%	8.6E−01	1.1%	0.4%	0.7%	**9.2E**−**09**
**19**	0.7%	1.1%	−0.5%	2.7E−01	0.8%	0.7%	0.0%	0.8%	0.8%	−0.1%	8.0E−01	0.8%	0.3%	0.5%	**1.6E**−**07**
**20**	0.5%	0.9%	−0.5%	2.4E−01	0.6%	0.5%	0.1%	0.6%	0.6%	0.0%	8.4E−01	0.6%	0.2%	0.4%	**1.3E**−**07**
**>20**	2.3%	3.7%	−1.4%	4.5E−01	2.4%	2.0%	0.5%	2.4%	3.4%	−1.0%	3.7E−01	3.1%	1.6%	1.4%	**9.4E**−**03**

In the whole genomes and both the coding and non-coding sequences of all the angiosperm species, the abundance of microsatellites decreased significantly as the motif repeat number increased, for all mono to hexanucleotide repeats (Table S3D).

It should also be noted that the distribution of microsatellites with respect to motif repeat number in the coding and non-coding sequences of all the angiosperm species showed great difference (Figure 5). Compared to the non-coding sequences, the average abundance of microsatellites in the coding sequences of all the angiosperm species was significantly higher for the 4–5 times of motif repeat but lower for the 3 and >6 times of motif repeat (Table S3E). In addition, the correlations between the abundance of microsatellites of the corresponding motif repeat numbers in the coding and non-coding sequences of the angiosperm species were generally moderate (mean r = 0.71 and 0.59 for the *Monocotyledoneae* and *Dicotyledoneae* species, respectively; Table S3F).

### Genomic Distribution of Microsatellites in Sequenced *Brassica* and Other Angiosperm Species

**Figure 6:**
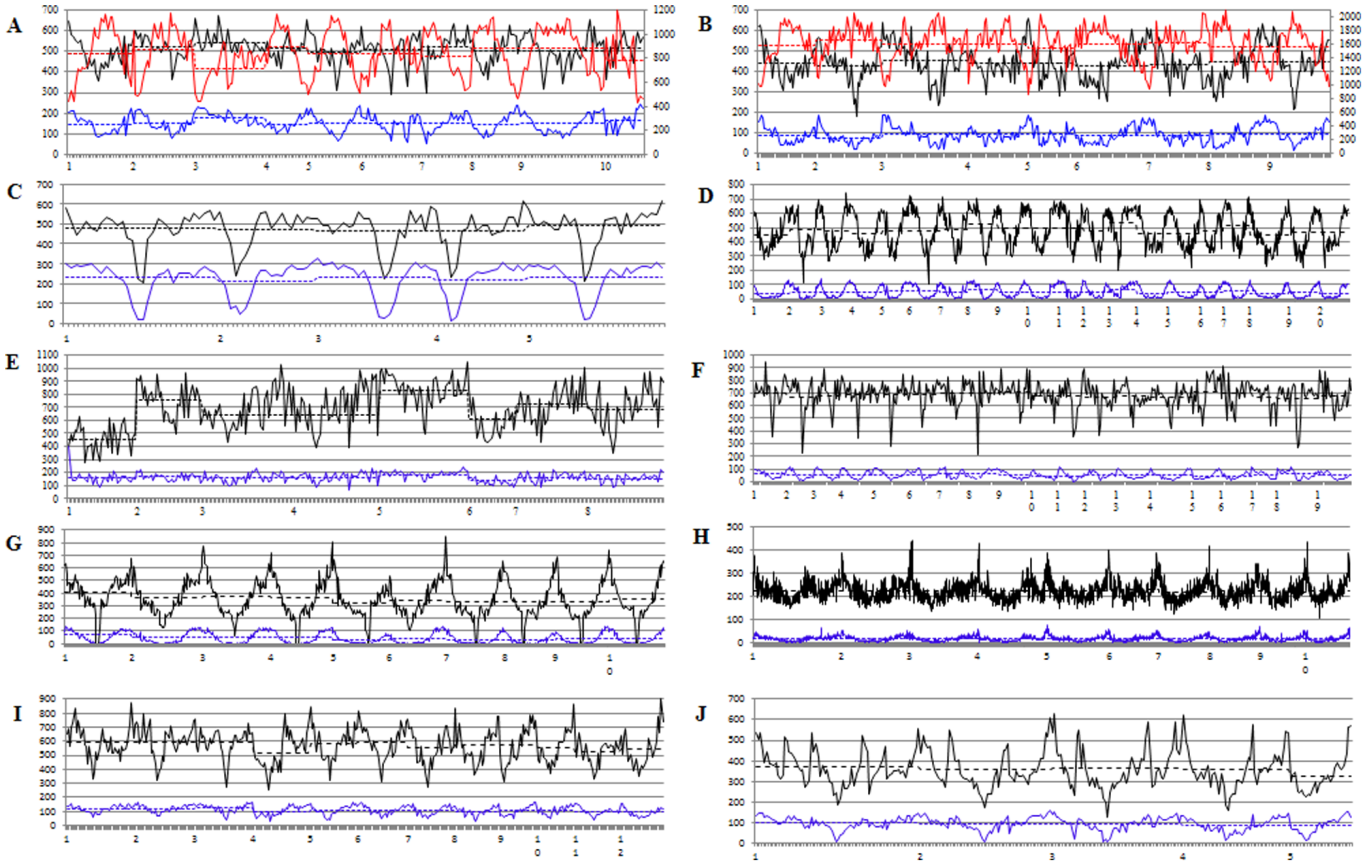
Genomic distribution of microsatellites as well as genes and TEs in the assembled pseudochromosomes of several sequenced angiosperm species, i.e., *B. rapa* (A), *B. oleracea* (B), *A. thaliana* (C), *G. max* (D), *M. truncatula* (E), *V. vinifera* (F), *S. bicolor* (G), *Z. mays* (H), *O. sativa* (I) and *B. distachyon* (J). The horizontal axis shows the assembled pseudochromosomes, which were divided into 1-Mb intervals. The left and right vertical-axis shows the frequency of microsatellites/genes and TEs, respectively. On the figure: the lines of different colors represent the distribution of microsatellites (black), genes (blue) and TEs (red), respectively; the lines of different types represent actual (solid) and hypothetical/even (dashed) distribution, respectively.

The genomic distribution of microsatellites and its relationship with annotated genomic components (mainly genes and TEs) were analyzed for ten angiosperm species (Figure 6) because of the availability of the assembled pseudochromosomes (http://www.phytozome.net; http://genomevolution.org/wiki/index.php/Sequenced_plant_genomes).

The average frequencies of microsatellites on the different chromosomes of the ten angiosperm species might be very similar (*A. thaliana* and *Z. mays*), generally comparable (*B. oleracea*, *V. vinifera*, *B. rapa*, *B. distachyon*, *O. sativa*, *G. max* and *S. bicolor*), or significantly different (*M. truncatula*). Obviously, the genomic distribution of microsatellites was highly uneven (Figure 6), which was consistent with the high significance of P-value of χ^2^ tests between their practical and hypothetical/average frequencies in the 1-Mb genomic intervals (Table 6). Typically, the frequency of microsatellite was high at the ends but low in/near the middle of all the chromosomes of the ten angiosperm species (Figure 6).

**Table 6:** P-value of χ^2^ test between the practical and hypothetical/average frequency of microsatellites and its correlation with genes and TEs within 1-Mb genomic intervals for the ten sequenced angiosperm species with available pseudochromosomes.

Species	P_χ2-test_	r_G_	r_T_
*B. distachyon*	2.3E−117	0.79	/
*O. sativa*	3.6E−57	0.73	/
*S. bicolor*	0.0E+00	0.9	/
*Z. mays*	2.0E−161	0.68	/
*B. rapa*	1.8E−14	0.72	−0.63
*B. oleracea*	6.9E−60	0.88	−0.73
*A. thaliana*	3.3E−57	0.85	/
*V. vinifera*	1.3E−22	0.44	/
*G. max*	1.1E−177	0.89	/
*M. truncatula*	1.2E−90	0.69	/

Interestingly, the general trend of the genomic distribution of microsatellites was basically accordant with that of genes but contrary with that of TEs in all the chromosomes of the ten analyzed angiosperm species (Figure 6), which was consistent with the significantly positive or negative correlation between the frequencies of microsatellites and genes (mean r = 0.76) or TEs (mean r = −0.68) respectively, in the 1-Mb genomic intervals studied (Table 6).

## Discussion

### Different Patterns of Microsatellite Distribution in Different Genomic Regions

Consistent with the generally low correlation for the microsatellite frequency or distribution with respect to motif length, type and repeat number in the coding and non-coding sequences (Table S1–3), these microsatellite characteristics of the angiosperm species displayed considerable differences between the two regions (Figure 2–5). Typically, these microsatellite characteristics were more conservative in the coding than non-coding sequences, especially for closely related species. In addition, these microsatellite characteristics of the angiosperm species also displayed significant differences according to genic region (e.g., untranslated regions, CDS and introns) (manuscript in preparation). More importantly, the physical distribution of microsatellites in different genomic regions (such as ends and middles of the chromosomes) was also highly nonuniform (Figure 6). In fact, similar results were also found in other studies that investigated the microsatellite frequency and distribution with respect to motif length and type in the different genomic/genic regions of several model and crop species [8], [9], [10], [14], [25]. These results strongly indicated that different patterns of microsatellite distribution across genomic regions exist and may be due to the different selective pressures acting on the microsatellites in different genomic regions [2], [4] owing to their different biological functions [1].

### Evolutionary Dynamics of Microsatellite Distribution in Polyploidy

The average frequency of microsatellites in the coding sequences of *A. thaliana* and *A. lyrata* was significantly lower than that of *B. rapa* and *B. oleracea*, and both were also slightly lower than that of *B. napus* (Table 1–2). This was consistent with previous findings: the frequencies of microsatellites in the transcribed sequences/unigenes of *A. thaliana*, *B. rapa* and *B. oleracea* were lower than that of *B. napus* [14], [26]; the duplicated genes in *Arabidopsis* typically contained a higher frequency of microsatellites [10]. These results strongly suggested that polyploidy may lead to the slight increase in the frequency of microsatellites in the coding sequences, which may be advantageous for evolution because microsatellites in coding sequences can be directly linked to gene function, providing a basis for quick adaptations to environmental changes [1], [4], [27]. Whereas, the average frequency of microsatellites in the whole genome/non-coding sequences of *A. thaliana* and *A. lyrata* species was slightly greater than that of *B. rapa* and *B. oleracea* (this difference was much larger when the frequencies were calculated from the true total genome sizes of the four species, data not shown), and both were also greater than that of *B. napus* (Table 1–2). This suggested that polyploidy may lead to the significant decrease in the frequency of microsatellites in the whole genome/non-coding sequences, which corresponds to the negative correlation between microsatellite frequency and both genome/non-coding sequences size and TEs content observed in this and other studies [8], [25]. This result is reasonable as polyploidy is often accompanied by the proliferation [28], [29], [30], [31] of TEs (which rarely contain microsatellites [25] and show a tendency to insert into some microsatellites, such as AT-rich repeats [32], [33]), the loss [20], [34], [35], [36], [37], [38] of genes (those are rich in microsatellites [25]), and the direct elimination [39], [40], [41], [42], [43], [44] of some microsatellites; these genomic changes can thus lead to a significant decrease in the frequency of microsatellites.

The distributions of microsatellites with respect to motif length, type and repeat number in the whole genome and the non-coding sequences and specifically within the coding sequences of *B. napus* were virtually identical to that of *B. rapa*/*B. oleracea*, and both were also highly similar to that of *A. thaliana*/*A. lyrata* (Figure 3–5), which was consistent with the high correlation coefficients between these variables (Table S1–3). This indicated that polyploidy, especially that involving recently occurring genome-duplication events (e.g., represented by *B. napus* vs. *B. rapa*/*B. oleracea*), may not lead to a significant change in the distribution of microsatellite with respect to motif length, type and repeat number. It should be noted that the correlation coefficients between these variables of *B. napus* and *B. rapa*/*B. oleracea* were slightly higher than those between *B. rapa*/*B. oleracea* and *A. thaliana*/*A. lyrata* (Table S1–3), which corresponds to the divergence time of these species (i.e., the divergence time between *B. napus* and *B. rapa*/*B. oleracea* is later than that between *B. rapa*/*B. oleracea* and *A. thaliana*/*A. lyrata*).

### Evolutionary Dynamics of Microsatellite Distribution may be Generally Consistent with the Plant Divergence/Evolution

For the species of same genus, their microsatellite characteristics (e.g., frequency and distribution with respect to motif length, type and repeat number) were highly similar (Figure 2–5; Table 1; Table S1–3). High similarity was also observed for several characteristics of microsatellites investigated in the EST sequences of three *Brassica* genus species [45] and in the genomic sequences of two *O. sativa* subspecies [46]. However, for the species of different genera, families, orders and classes (e.g., *Brassica* vs. *Arabidopsis*, *Brassicaceae* vs. *Caricaceae*, *Brassicales* vs *Fabales* and *Monocotyledoneae* vs. *Dicotyledoneae*), the differences in their microsatellite characteristics usually become larger (Table 2, 3, 5; Table S2–3C). Similar results were observed in studies of several characteristics of microsatellites in the UTR/CDS sequences of ten species from the *Brassicaceae*, *Solanaceae* and *Poaceae* families [14], the genomic/EST sequences of eight species from the *Monocotyledoneae* and *Dicotyledoneae* classes [8], the genomic/CDS sequences of six species from the *Monocotyledoneae* and *Dicotyledoneae* classes [9], and the EST sequences of eleven species from the *Angiospermae*, *Gymnospermae*, *Bryophyta*, *Pteridophyta* and *Chlorophyta* phyla [15]. These results indicated that the pattern of microsatellite distribution may be generally accordant with the divergence/evolution of plants. This is understandable because microsatellites are one of the three major classes of genetic variations and have many important biological functions [1], [2], [4] and increasing evidence has demonstrated that variations in microsatellites may lead to phenotypic variations [47], [48], [49] and adaptive evolution [50], [51].

### Dichotomous Evolutionary Pattern of Microsatellite Distribution in Angiosperms

Interestingly, by comparing these microsatellite characteristics in both the whole genomes and specific genomic regions (such as coding and non-coding sequences) all analyzed angiosperm species naturally diverged into two clearly different groups according to monocot or dicot classification (aside from a few exceptional species).

First, the average frequencies of microsatellites in the whole genomes, the non-coding sequences and especially in the coding sequences of the monocots and dicots were significantly different (Figure 2; Table 1–2). Compare to the monocots, the average microsatellite frequency of the dicots was slightly higher for the whole genome and the non-coding sequences but much lower for the coding sequences. This indicated that different patterns of selective pressures acted on the microsatellites in the whole genome and both the coding and non-coding sequences of monocots and dicots (i.e., the selective pressures acting on the microsatellites were much higher for the coding sequences and significantly lower for the whole genome and non-coding sequences of dicots versus monocots).

Second, the distributions of microsatellites with respect to motif length in the coding sequences and especially in the non-coding sequences and the whole genomes of the monocots and dicots (except for *L. usitatissimum*) were clearly different (Figure 3; Table 3). Compared to the monocots, the average abundances of microsatellites in the whole genomes and the non-coding sequences of the dicots were greater for mono- to dinucleotide repeats, but less for tri- to hexanucleotide repeats, indicating that shorter motifs may be subjected to stronger selective pressure in monocots than dicots. Theoretically, shorter motifs allow for more potential replication slippage events per unit length of DNA [52] and are thus likely to be more unstable and carry higher mutation rates [53], [54]. Therefore, our results also suggested that the microsatellite mutation rates may be higher in dicots than monocots, which is in accordance with previous experimental estimations of mutation rates in several dicots [52], [55] and monocots [56]. Due to the triplet nature of codons, the trinucleotide repeat was dominant in the coding sequences of all the angiosperm species (Figure 3; Table 3). Compared to the monocots, the average abundance of microsatellites in the coding sequences of the dicots was lower for tri- and hexanucleotide repeats but higher for the other four types of microsatellite repeats, which suggested a preference for fewer frame-shift mutations in the microsatellites of monocots than dicots.

Third, the distributions of microsatellites with respect to motif type (especially the dominant/major and absent/scarce motifs) in the whole genomes and both the coding and non-coding sequences of the monocots and dicots (except for *L. usitatissimum*) were clearly different (Figure 4; Table 4; Table S2C). Although the relative A/T or C/G contents in the analyzed sequences (Table 1) corresponded well with the nucleotide composition characteristics (rich in A/T or C/G) of the motifs those were dominant/major, absent/scarce (Table 4) or with significantly different abundances between monocots and dicots (Table S2C) or between coding and non-coding sequences (Table S2D), they are not large enough to explain the variations in the abundances of all types of motifs in all analyzed angiosperm species [8], [9]. For example, the abundance of many motifs exhibited great variation between species with similar A/T or C/G contents (e.g., 228.7-fold difference in the relative abundance of AGCCTC in *M. esculenta* and *M. guttatus*), the dominant/major or absent/scarce motifs were generally not fully comprised of A/T or C/G sequences (e.g., AG, AAG, AAAG, AAAAG and AAGAGG were the dominant/major motifs in the coding sequences of dicots), and the practical proportions of the motifs with theoretically equal abundances (e.g., AC and AG) were found to differ across all analyzed angiosperms. Therefore, the different structures and functions of the various motifs [1], [2], [5], the different selective pressures acting on the specific motifs in different species [9] and/or other unknown mechanisms may also be responsible for the observed variations in motif abundance in plants.

Fourth, the distributions of microsatellites with respect to motif repeat number in the coding sequences and especially in the non-coding sequences and the whole genome of monocots and dicots (except for *L. usitatissimum*) were also significantly different (Figure 5; Table 5; Table S3C). Compared to the monocots, the abundances of microsatellites in the whole genome and both the coding and non-coding sequences of the dicots were lower for the smaller motif repeat numbers but higher for the larger motif repeat numbers, suggesting that the expansion of repeat motif may be subjected to stronger selective pressure in monocots than dicots.

More importantly, the correlation between the above-mentioned microsatellite characteristics in the coding and non-coding sequences of dicots was much lower than that of monocots. This strongly indicates that there are different patterns of selection pressures acting on microsatellites in the coding and non-coding sequences of monocots and dicots (i.e., the selection pressures acting on microsatellites in the coding and non-coding sequences are more similar in monocots than they are in dicots).

Taken together, these significant differences in so many microsatellite characteristics may imply a dichotomous evolutionary pattern of microsatellite distribution in angiosperms because their typical representatives, monocots and dicots, diverged from a common ancestor approximately 200 MYA [22]. Further investigation is required to determine which pattern is more or equally advantageous for evolution. However, it should be noted that certain microsatellite characteristics of a few analyzed angiosperms did not correspond to their phylogenetic classification (e.g., the distribution of microsatellites with respect to motif length in the whole genome/non-coding sequences of the dicot species *L. usitatissimum* was more similar to that of monocots, whereas the ratio of microsatellite frequency in the non-coding and coding sequences of this species was between that observed for monocots and dicots), which strongly indicated the complexity of the evolutionary pattern of microsatellite distribution.

### Constant Microsatellite Characteristics in Plant Evolution

The current investigation also revealed several constant microsatellite characteristics in plant evolution, as the observed high level of consistency among all the species investigated in this and other studies [8], [9], [14], [15], [25], [45], [57], [58], [59] was not likely a chance event. First, trinucleotide repeat microsatellites were dominant in coding sequences (Figure 3; Table 3), which is undoubtedly caused by the triplet nature of codons [4]. Second, microsatellite abundance decreased as the motif length, motif repeat number, and repeat length (i.e., motif length × motif repeat number) increased (Table S3D), which may be explained by longer repeats having higher mutation rates and the potential to produce instability [60]. Third, the microsatellite frequency and distribution with respect to length, type and repeat number of motifs seemed to be more conservative in the coding than non-coding sequences (Figure 2–5; Table S1–3B), which is likely caused by the functional importance of coding DNA sequences. Fourth, microsatellite frequency was high at both terminals and low in/near the middle of each chromosome (Figure 6), which likely corresponds to the telomeric and peri-centromeric regions, respectively [61]. In addition, the general trend of the genomic distribution of microsatellites was basically accordant with that of genes but contrary with that of TEs (Figure 6; Table 6), which is in agreement with previous findings showing that microsatellites are preferentially associated with non-repetitive DNA sequences/genes in plant genomes [2], [25]. It should be noted that all of these constant microsatellite distribution characteristics (such as the dominance of trinucleotide repeat in the coding sequences) can be explained by the general biological rules (such as the triplet nature of codon).

## Materials and Methods

### Genome Sequences of the Sequenced *Brassica* and Other Angiosperm Species

Based on cooperative efforts from several institutes, including our own, the genomes of *Brassica rapa* cultivar Chiifu-401-42 [16], *Brassica oleracea* cultivar O212 (submitted) and *Brassica napus* cultivar Zhongshuang no.11 (our unpublished data) were sequenced using Illumina GA II technology, and high-quality sequence reads were assembled using stringent parameters (http://www.brassica.info/resource/sequencing.php). To study the evolutionary dynamics of microsatellites distribution in plants, the genome sequences of other sequenced angiosperm species were downloaded from the Phytozome (http://www.phytozome.net) and Cogepedia (http://genomevolution.org/wiki/index.php/Sequenced_plant_genomes) websites, from which the phylogenetic trees of these sequenced plants were also obtained. The detailed classification grades of the sequenced *Brassica* and other angiosperm species were identified by the NCBI Taxonomy Browser (http://www.ncbi.nlm.nih.gov/Taxonomy/taxonomyhome.html).

### Identification of Microsatellites

PERL5 script MIcroSAtellite (http://pgrc.ipk-gatersleben.de/misa/) [62] was used to identify perfect microsatellites in the whole genome and both the coding and non-coding DNA sequences of the sequenced *Brassica* and other angiosperm species. To identify the presence of microsatellites, only 1- to 6-nucleotide motifs were considered because microsatellites with longer motifs are very scarce. The criteria for microsatellite selection were as follows: mononucleotide, ≥12 repeats; dinucleotide, ≥6 repeats; trinucleotide, ≥4 repeats; and tetra- to hexanucleotide, ≥3 repeats.

### Statistical Analysis

The correlation analysis was performed using the SAS PROC CORR procedure incorporated in the SAS v8.0 software package [63]. The Excel statistical function CHISQ.TEST was used to obtain the significance level (P_χ2-test_) of the degree of fit for the practical and hypothetical distributions of microsatellites as well as genes and TEs in the assembled pseudochromosomes. The Excel statistical function T.TEST was used to obtain the significance level (P_t-test_) of the differences in microsatellite frequency and abundance between the coding and non-coding sequences and between different genera, families, orders or classes.

## Supporting Information

Table S1 The correlation and difference for the abundance of corresponding mono- to hexanucleotide repeat microsatellites in the whole genomes and both the coding and non-coding sequences of the analyzed angiosperm species.
(XLS)

Table S2 The correlation and difference for the abundance of the corresponding mono- to hexanucleotide motifs microsatellites in the whole genomes and both the coding and non-coding sequences of the analyzed angiosperm species.
(XLS)

Table S3 The correlation and difference for the abundance of the corresponding motif repeat numbers microsatellites in the whole genomes and both the coding and non-coding sequences of the analyzed angiosperm species.
(XLS)
